# HIV Disclosure Anxiety: A Systematic Review and Theoretical Synthesis

**DOI:** 10.1007/s10461-016-1453-3

**Published:** 2016-07-12

**Authors:** Michael Evangeli, Abigail L. Wroe

**Affiliations:** Department of Psychology, Royal Holloway University of London, Egham, Surrey TW20 0EX UK

**Keywords:** HIV disclosure, Anxiety, Fear, Cognitive, Model

## Abstract

HIV disclosure can help people living with HIV to access social support, enhance antiretroviral adherence, facilitate engagement in care and reduce unprotected sex. Given interpersonal risks associated with HIV disclosure, however, anxiety about sharing one’s status is common. To investigate anxiety about HIV disclosure in HIV-positive populations, we conducted a systematic review of qualitative and quantitative studies, with 119 studies included. The review demonstrated that perceived interpersonal risks are associated with HIV disclosure and outlined evidence of associations with anxiety, fear and worry. We present a new cognitive model of HIV disclosure anxiety adapted from clinical theories of health and social anxiety, consistent with evidence from the review. The model attempts to explain the development and maintenance of anxiety in individuals whose functioning is *most* affected by concerns about sharing their status. Implications for helping people living with HIV struggling with significant levels of anxiety about HIV disclosure are discussed.

**Electronic supplementary material:**

The online version of this article (doi:10.1007/s10461-016-1453-3) contains supplementary material, which is available to authorized users.

## Introduction

HIV disclosure (sharing one’s HIV positive status with others) has a number of potentially positive consequences. HIV partner disclosure can reduce levels of unprotected sexual activity, partly through greater condom negotiation and use [1–3], and facilitate partner HIV testing [1]. Sharing one’s status to partners or others can improve engagement in care [1, 4], and help in the initiation of and adherence to antiretroviral treatment (ART) [5], through the availability of disclosure-specific support or the reduced need to hide medication from others [6]. There may also be psychological benefits to status sharing. Well-being may be enhanced (although evidence is equivocal [7]) through increased social support [8], the development of more helpful cognitive appraisal of HIV-related stressors and enhanced self-esteem [9, 10]. HIV disclosure may reduce anxiety levels, although again, evidence is equivocal [1, 7].

Given the potential individual and public health benefits associated with HIV disclosure, the process of sharing one’s status with others could be thought of as a helpful health behaviour. HIV disclosure, however, exposes the person living with HIV (PLHIV) to potential rejection and discrimination. This is the case whether HIV disclosure is direct (the PLHIV telling others about their status), indirect (somebody else revealing the PLHIV’s status to others), or guessed (others concluding that the PLHIV is HIV-positive) [11]. In addition, once one’s status is shared with a particular individual, it cannot be *un*shared. Given the interpersonal risks associated with HIV disclosure, *anxiety* about sharing one’s status is likely to be the norm, generally serving to inhibit HIV disclosure. Concerns about other people knowing one’s HIV status may be heightened to the extent that very little or no HIV disclosure takes place. This can contribute to feelings of social isolation [12]. There may be some situations, however, when anxiety is influential in motivating disclosure rather than non-disclosure (e.g., when the PLHIV is concerned about others finding out about one’s status from a third party).

Some models of health behaviour recognise the importance of anxiety. The Self-Regulatory Model of Illness Behaviour [13] suggests that fear and anxiety (amongst other emotional and cognitive factors) influences coping with health threats. Fear is also included in the Protection Motivation Theory [14] as a factor that influences behavioural intentions and health behaviour.

To investigate the nature of anxiety about HIV disclosure in HIV-positive populations, we conducted a systematic review. As rates of HIV disclosure (and potentially the nature of HIV disclosure anxiety) differ according to context (e.g., the characteristics of the person living with HIV, the disclosure recipient, route of infection, whether disclosure is direct, indirect or guessed, and the time since diagnosis [11, 15, 16]), our review adopted an inclusive approach to study eligibility.

## Method

### Study Eligibility Criteria

Studies were included in the review if they:

1. Were empirical, reporting primary data;
2. Included HIV-positive participants;
3. Reported or measured anxiety, worry or fear about HIV disclosure or one’s HIV status being shared;
4. Referred to the PLHIV sharing their HIV status with others, others sharing the PLHIV’s status with third parties, or others guessing the PLHIV’s status;
5. Assessed the association between anxiety/worry/fear about sharing one’s HIV status and any outcome, or cited disclosure anxiety/worry/fear as a reason for non-disclosure or any other outcome.

### Sources of Information and Search Strategy

Studies published in peer-reviewed journals were retrieved from the electronic databases Pubmed/Medline and PsychINFO. There was no date restriction. The searches were conducted using combinations of the following terms: ‘anxiety’, ‘fear’, ‘worry’ and ‘HIV disclosure’ in either the title or abstract. The searches were conducted on 2nd January 2015.

### Data Collection and Abstraction

The first author carried out the initial searches. All duplications were removed. The first author screened the remaining titles and abstracts for eligibility. A random sample of 20 % of the articles at this stage was independently rated by both the first author and a second reviewer (an undergraduate psychology student). Inter-rater reliability was calculated using Cohen’s Kappa. The value for Cohen’s Kappa was 0.78, *p* < 0.01, suggesting a good level of inter-rater reliability. Articles considered relevant were retrieved in full text. These articles were then assessed for eligibility by both reviewers. Exclusions were reported, with reasons given.

Data was extracted for the following study characteristics (by the undergraduate psychology student, verified by the first author): authors; date of publication; location; design; methodology; nature of the sample including sex and age; nature of disclosure recipient; and findings relating to HIV disclosure anxiety.

## Results

After duplicates were removed, there were 426 articles. 159 articles were screened in as potentially eligible. 119 of these articles were subsequently considered eligible for the review. Most commonly articles were rejected as they did not report on anxiety *about HIV disclosure*.

**Fig. 1:**
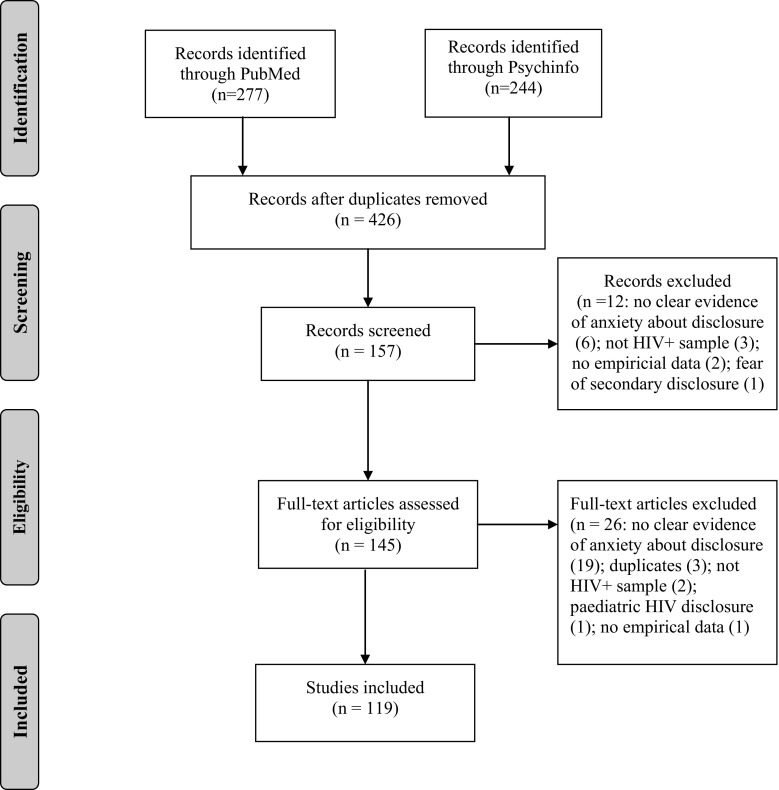
Study search process. *From*: Moher et al. [46]

Figure 1 summarises the review process.

Study characteristics are summarised in Supplementary Table 1.

### Study Characteristics

Fifty-one of the studies took place in Africa, with a further 39 studies in North America. Fifteen studies took place in Asia, with 8 in Europe, 4 in South America and 2 in Australasia. There were three intervention studies [17–19]. The remainder of the studies were cross sectional.

The most commonly used method to capture data was qualitative interviews (64 studies). Surveys were used in 43 studies, with focus groups in 18 studies. The other approaches used were self-report questionnaires (8 studies) and participant observations (3 studies). Fifteen of the studies used more than one method.

### Participants and Disclosure Recipients

Sample sizes ranged from four to 775 (median sample size 52, IQR 26–164). Most commonly (59 studies), participants did not belong to a specific subgroup of HIV-positive individuals. Twenty-three studies were based on parent samples. Other studies sampled ethnic minority groups (eight studies), individuals taking ART (six studies), adolescents (five studies), and MSM (three studies). The remainder (15 studies) sampled from diverse populations (e.g., intervention participants, sex workers and prisoners). Seventy-seven of the studies sampled both males and females, 32 accessed female only samples, and ten studies sampled males only. The disclosure recipient was not specified in 77 of the studies. HIV disclosure to one’s partner was the focus of 24 studies. Other studies focused on disclosure to children (nine studies), family and friends (five studies), work colleagues (three studies) and dentists (one study).

### Qualitative and Survey Findings

The most commonly reported reason for non-disclosure or disclosure anxiety theme was the fear of discrimination or stigmatising responses from others (58 studies). Anxiety about rejection and abandonment (e.g., divorce from partner) was also reported frequently (54 studies) with the specific fear of partner violence cited in 18 studies. Anxiety about secondary/indirect disclosure (disclosure recipients sharing one’s status with others) was commonly reported (17 studies), as was the concern about causing stress, worry and burden to others if they were disclosed to (15 studies). There were a range of other disclosure anxiety themes reported, including the fear of being blamed, worries about being isolated, anxiety about feeling guilty and ashamed, and concerns about losing one’s job.

Anxiety about HIV disclosure was reported to be a barrier to ART initiation or adherence in 14 studies, and to engagement in care for oneself or one’s child in eight studies. HIV disclosure anxiety was given as a reason for being isolated or lacking social support in five studies and was mentioned as a barrier to safer sex (e.g., condom use) in three studies.

### Quantitative Findings

A range of statistical associations with anxiety about HIV disclosure were reported in eight studies. Most commonly, associations with psychological variables were assessed. ‘HIV disclosure concerns’ was related to higher levels of general anxiety in two studies [20, 21]. One study found a statistically significant relationship between ‘HIV disclosure concerns’ and depression [21], but a different study failed to find this association [20]. The latter study also failed to find an association between ‘HIV disclosure concerns’ and life satisfaction but did find an association with reduced social support [20]. ‘HIV disclosure concerns’ were associated with higher levels of HIV stigma in two studies [21, 22], and were associated with negative self-image, greater disengagement coping, less time since diagnosis, less primary control engagement and lower self-esteem in one study [21].

In relation to demographic and clinical variables, ‘HIV disclosure concerns’ was greater in females compared to males in one study [23], and in heterosexual compared with homosexual/bisexual participants in three studies [23–25]. ‘HIV disclosure concerns’ were greater in individuals without an AIDS diagnosis in two studies [24, 25], and greater in those with lower CD4 counts, no HIV-related hospitalisations, no partner, and of younger age in one study [25].

## Discussion

The review suggests that anxiety about disclosing one’s HIV status is highly prevalent across region and population. Fears about rejection and discrimination from others appear ubiquitous. More generally, anxiety seemed to focus on the possibility of negative effects for the individual living with HIV in the context of interpersonal relationships. There were also, however, frequently reported concerns about the effect on others of sharing one’s HIV status. There is some evidence from qualitative studies included in the review that negative consequences of anxiety about HIV disclosure, in terms of reduced ART adherence, poor engagement in care, and isolation, may occur. Importantly, participants themselves often cited fears about HIV disclosure as a reason for such negative outcomes. No quantitative studies examined these relationships.

The review was limited by the fact that most of the studies were qualitative in nature. In addition, the few quantitative studies were cross sectional with only a limited range of correlates examined. Hence, it was not possible to quantify the extent to which HIV disclosure anxiety is related to other psychological and behavioural factors or to examine the causes and consequences of HIV disclosure anxiety from the studies. Further, the majority of studies did not specify to whom the person living with HIV was disclosing. This is important as disclosure anxiety may differ depending on the recipient.

### Models of HIV Disclosure

There is a long tradition of developing models of HIV disclosure separate from models of the disclosure of other health conditions [26]. This may reflect specific issues associated with HIV (e.g., HIV stigma) and the fact that HIV disclosure may facilitate reductions in onward transmission. Given the likely importance of anxiety about disclosing one’s HIV status, it is surprising that explanatory models of HIV disclosure have rarely included anxiety as a central construct. A recent systematic review of HIV disclosure models [27] found that individual cognitive determinants of HIV disclosure were commonly cited in models (e.g., self-efficacy and perceived disclosure risks and benefits), but affect, including anxiety, was not. Indeed, neither the presence of anxiety *nor how it is develops and can be maintained* has been included in HIV disclosure theories. Not all disclosure decisions are likely to be influenced by anxiety but our systematic review suggestions that anxiety about HIV disclosure is common and may have important consequences. We suggest that *high* and *persistent* levels of anxiety about disclosure may have an impact on an individual’s quality of life and potentially their social and occupational functioning.

A new theoretical model of HIV disclosure anxiety (rather than of disclosure *behaviour*) may help to direct clinicians towards interventions that help to support people with HIV with significant levels of anxiety and preoccupation about sharing their status with others. This may be particularly important where anxiety about HIV disclosure is having a large impact on the individual’s psychological and relational functioning and their engagement with care.

### Aims of Our Model

We present a new model of HIV disclosure anxiety that draws upon existing cognitive models of anxiety disorders to explain how high levels of anxiety about sharing one’s status with others develops and can be maintained. The approach of drawing on theoretical models relating to affect rather than of heath behaviour is novel in the HIV context. For example, the Health Belief Model [28] was the most commonly used model used in a recent review of psychological correlates of HIV testing [29], and the Information Motivation Behavioural Skills Model [30] (based on the Theory of Planned Behaviour [31]) has guided much recent research on antiretroviral adherence. Such models primarily outline *cognitive* determinants of *behaviour*. It may be, however, that *affect* is another important determinant of many HIV-related behaviours. For example, consistent relationships between fear of HIV and HIV testing on the one hand and HIV testing itself on the other hand, have been reported [29]. It may also be that affect (particularly anxious affect) plays a key role in driving many HIV disclosure decisions. Of greater relevance for our model, however, is the possibility that high levels of anxiety about disclosure affects individuals’ quality of life and their social and occupational functioning (through processes such as avoidance leading to social isolation).

Existing cognitive models of anxiety disorders emphasise cognitive and behavioural factors that *maintain* anxiety and minimise quality of life. Models of *social* anxiety [32] are particularly relevant as HIV disclosure is a social phenomenon. Models of *health* anxiety [33] are also relevant given the nature of the condition and the context of care and treatment. Such cognitive models attempt to integrate cognitive, affective and behavioural factors and have clear treatment implications. Existing cognitive models of anxiety disorders cannot be applied in their entirety, however. There are specific characteristics associated with HIV that require an HIV-specific model, including the extent of HIV stigma, and the infectious nature of the condition. In addition, models of social anxiety [32] have self-focused attention at their core, whereas our systematic review suggests that it is the fear of the response of others which is central to HIV disclosure anxiety. Models of health anxiety [33] also suggest that self-directed interpretations (of symptoms) are key rather than interpersonal factors.

Our model is designed to apply to individuals who have high levels of anxiety about HIV disclosure (e.g. a persistent fear of disclosure, with intense anxiety about one’s status being known) that has a *significant* impact on their functioning (e.g., significant interference with their normal routine, occupational/academic functioning, or social activities or relationships). This may be manifested in individuals being preoccupied with thoughts about not telling others that they are HIV-positive (direct disclosure), or about avoiding people finding out that they are HIV positive (indirect or guessed disclosure). The model is intended to apply to situations where the PLHIV fears their HIV-positive status will be shared (i.e., where they are motivated to avoid disclosure) or to situations where they want to or feel they have to disclose but are anxious about doing so (i.e., where to some extent the PLHIV is motivated to disclose). That is, anxiety is only likely to characterise situations where there is a level of motivation to *either* disclose or to avoid disclosure.

Our model attempts to explain why some individuals are more affected by anxiety about sharing their status than others and why some situations are more anxiety-provoking than others, despite the ubiquitous nature of HIV disclosure anxiety. We argue, however, that levels of HIV disclosure anxiety are on a continuum both between people and within people (depending on the disclosure recipient and over time). At lower levels of anxiety about HIV disclosure, this may not interfere with decisions about sharing one’s status and may have a minimal impact on quality of life. The model aims to be consistent with existing evidence about HIV disclosure anxiety and HIV disclosure rates.

### A Model of HIV Disclosure Anxiety

**Fig. 2:**
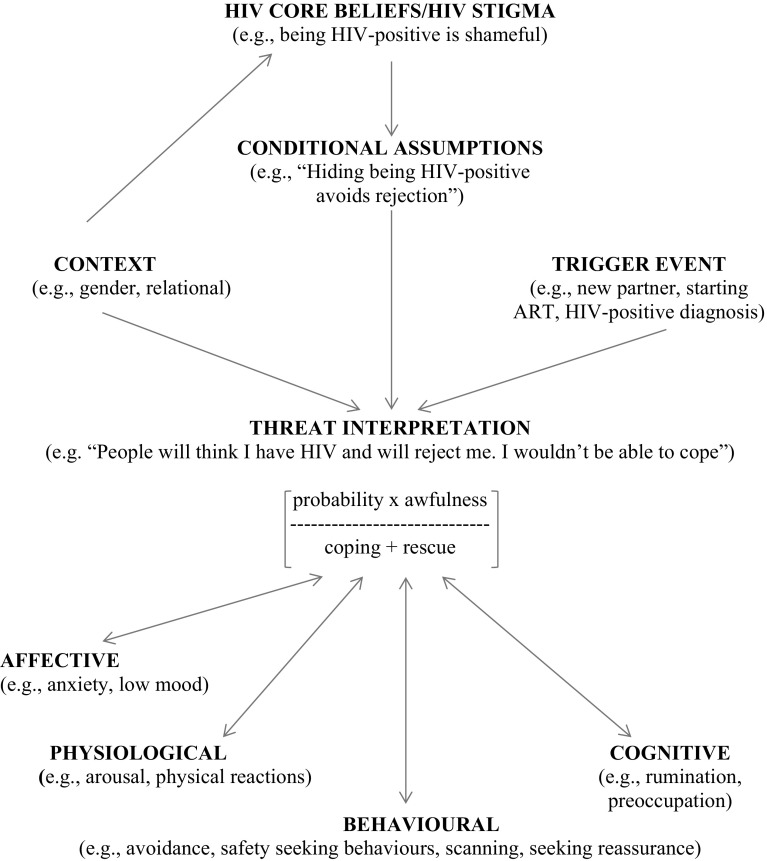
A model of HIV disclosure anxiety

The model is outlined in Fig. 2.

Evidence in relation to the model is presented in Table 1.

**Table 1:** Evidence for model components

Model component	Sub-component	Evidence
HIV core beliefs/HIV stigma		Higher levels of HIV stigma associated with higher levels of HIV disclosure concerns [21, 22] and lower rates of HIV disclosure [12]; various studies reported in [8]
Conditional assumptions		No evidence
Context		Greater disclosure concerns in women [23] and heterosexual samples [23–25]. More disclosure fear in those with no income [39]. Different patterns of disclosure in males and females, and in marital and non-marital relationships [39]. More partner disclosure associated with better relationship quality [42]
Trigger event		Various studies report anxiety about HIV disclosure in relation to ART initiation (e.g., [47]), ART adherence (e.g., [48]), engagement in care (e.g., [49]), and safer sex [50]
Threat interpretation		Many examples of perceived threat associated with HIV disclosure. No evidence of hypothesized components of threat interpretation
Maintaining factors	Behavioural	Hiding of bottle feeding [45], only disclosing to other HIV+ people [12], using condoms instead of disclosing [44], avoiding sexual relationships [44], missing ART doses (e.g., [51]), not initiating ART (e.g., [47]), not attending clinic (e.g., [52]), and avoiding the use of condoms (e.g., [53])
	Cognitive	No evidence
	Affective and physiological	HIV disclosure concerns associated with anxiety [20, 21]

### HIV Core Beliefs/HIV Stigma and Conditional Assumptions

We suggest that internalised HIV stigma [34] (endorsing negative beliefs and feelings about HIV about oneself as an HIV-positive person) is a key distal determinant of HIV disclosure anxiety. Indeed, a relationship between HIV stigma and HIV disclosure concerns has been shown [21, 22], and there is evidence of an association between HIV stigma and reduced levels of HIV disclosure [8, 12]. Feelings of shame, perhaps based on actual experiences of discrimination after previous disclosure [12] (enacted stigma [34]) or difficulties in adjusting to an HIV-positive diagnosis, are argued to predispose individuals to HIV disclosure anxiety. Indeed, feelings of shame have been reported to inhibit HIV disclosure [35], and conversely self-compassion has been associated with HIV disclosure [36]. Negative beliefs about HIV (e.g., “People who are HIV-positive will have a short life expectancy”) are likely to contribute to internalised stigma. Illness beliefs are included as important determinants of health behaviour in many theoretical models [13] and have shown to be related to depression in HIV-positive individuals [37]. There may be broader core beliefs about illness (e.g., “people who are ill are to blame”) and the self (e.g., “I am not a strong person”) that impact upon HIV core beliefs.

Our model states that several contextual aspects are likely to influence the extent to which internalised HIV stigma is experienced and negative HIV core beliefs are endorsed. We use distinctions offered by Skovdal et al. [38]. Aspects of the *symbolic context* (e.g., community values, community HIV stigma, gender and sexuality representations) may impact upon internalised HIV stigma and HIV disclosure anxiety. Indeed, greater HIV disclosure concerns been reported in women [23], and heterosexual participants [23–25]. The *material context* may be relevant, consistent with evidence that greater disclosure fear is reported in those with no income [39]. Finally, *the relational context* is likely to influence internalised HIV stigma and HIV disclosure anxiety. For example, the HIV status of one’s partner and family members, their beliefs about HIV and HIV disclosure, and the quality of these relationships (e.g., the level of perceived trust), may influence internalised HIV stigma and HIV disclosure anxiety.

Cognitive models of depression [40] suggest that core beliefs produce conditional assumptions or rules that protect against distress and are activated in specific situations. For example, a belief that HIV is shameful may lead the individual to believe that they must hide their status from others to avoid being devastated by rejection (e.g., “If I hide my status, then I will be safe”). Such rules for living can, however, be unhelpful if they form a barrier to personally important goals (e.g., accessing support). Our model, therefore, includes the construct of conditional disclosure assumptions, arising from internalised HIV stigma.

### Trigger Event

We argue that HIV disclosure anxiety is heightened by specific events or situations which are interpreted in the light of negative core HIV beliefs and conditional assumptions about HIV disclosure. Indeed, our review suggested that HIV disclosure anxiety is present in particular contexts (e.g., taking ART medication in social situations, attending clinic, sexual situations) where HIV disclosure is thought to be required or wanted (direct disclosure), or where there might be a concern that one’s status will be revealed involuntarily or guessed (indirect or guessed disclosure). For example with the latter situation, individuals may be concerned that their antiretroviral use or their visits to clinic will be observed by others and that their status will, therefore, be inferred. An HIV diagnosis is likely to be an initial trigger to HIV disclosure anxiety but subsequently episodes of disclosure anxiety may be prompted by different determinants.

Within sexual relationships, a sense of responsibility to one’s partner and/or a concern about onward HIV transmission may trigger anxiety about disclosing, with a normative belief that one *should* share one’s status. This may occur in new relationships or when there is increasing depth within relationships. Within friendships, a desire for closeness may trigger a perceived need to disclosure. Such situations may trigger disclosure approach goals (e.g., sharing one’s status to enhance relationship quality) or disclosure avoidance goals (e.g., hiding one’s status to avoid rejection and reduce potential relationship conflict) [41].

### Threat Interpretation and HIV Disclosure Anxiety

A sense of threat (usually interpersonal in nature) is central to HIV disclosure anxiety. Our model uses a quasi-mathematical equation taken from a cognitive-behavioural model of health anxiety [33] to describe this threat interpretation: the probability of a negative outcome multiplied by the awfulness of the outcome, divided by coping plus rescue factors.

Greater anxiety will occur if the *probability* of negative outcomes (e.g., rejection from others) is predicted to be high. The greater the extent to which these outcomes are judged as *awful*, the more that anxiety is a likely outcome. Although these two synergistic components of perceived threat have not been separated in the HIV disclosure literature, it is likely that fears about discrimination/stigma, rejection, the effect on others and violence from studies included in the systematic review, are heightened by both perceptions of the likelihood and awfulness of the anticipated outcome. These perceptions may be experienced in the form of negative automatic thoughts or anxiety-laden images. *Coping* factors refer to the confidence in being able and prepared to face the anxiety of both sharing one’s HIV status and the outcome of HIV disclosure (and to cope with this anxiety). This may or may not be related to one’s *actual* ability to manage anxiety in the feared situation or to disclose effectively. Perceived coping ability (related to the concept of self-efficacy) is likely to predict the extent to which an individual is motivated to face and persist in challenging situations related to status sharing. *Rescue* refers to the perceived ability of others to minimise the individual’s anxiety about disclosing and to provide helpful support.

Previous disclosure experiences (and how these are appraised) are likely to influence all of the elements of the threat equation. Some theorists have, indeed, specified a role for previous disclosure experiences in making subsequent disclosure decisions [41] and there is evidence that negative disclosure experiences inhibit future HIV disclosure [12]. One of the consequences of not sharing one’s HIV status is that there is no opportunity for the person living with HIV to learn that others’ responses to HIV disclosure may be benign or supportive (or that they themselves can tolerate negative responses). As a result there may be an ongoing fear about disclosing, with less social support available if the individual chooses to share their status in the future [12].

Contextual factors will influence the extent to which the situation is perceived as threatening. *Relational* factors are argued to be particularly important. There is evidence that women living with HIV often disclose their status to family members first and then to partners, whereas men are more likely to disclose to partners first [39]. There is also evidence that married individuals are more likely to disclose to partners, whereas non-married individuals tend to share their status with their family [39]. Finally, the quality of intimate relationships is associated with partner disclosure occurrence [42]. These patterns of disclosure may signal differing levels of anxiety by characteristics of the discloser and their assessment of interpersonal threat associated with the disclosure recipient. Given the perceived threat associated with HIV disclosure, it is unsurprising that ambivalence about whether/when to disclose is experienced. Indeed, one study reports the fear of abandonment weighing against the need for support and the desire to raise risk awareness in HIV disclosure decisions [43].

### Maintaining Factors

Central to our model is the role of maintaining processes. It is these thoughts, feelings and behaviours that potentially maximise the negative impact of HIV disclosure anxiety and turn normative anxiety into something more problematic for the individual.

#### Behavioural Factors

The most powerful anxiety maintenance process is *avoidance* of situations that provoke anxiety (situations where one’s status may be shared or inferred). The individual may, for example, avoid telling others about being HIV-positive and as a result may use condoms in sexual relationships rather than disclosing, only take medication when alone, and avoid sexual relationships completely to avoid sharing one’s status [44]. It is important to note that avoidance is a self-protective strategy that is helpful in the short term (as it reduces anxiety). Avoidance, however, reinforces threat interpretations as the individual fails to disconfirm anxious predictions and they remain alert to future threats [33]. We include avoidance in our model to signal situations where widespread avoidance of disclosure anxiety-provoking situations is part of a maintaining cycle that has a negative impact on individual functioning (given the effects of, for example, ongoing anxiety and reduced social support).

Closely associated with avoidance is the use of *safety seeking behaviours* [32]. These are behaviours intended to prevent or minimise feared negative outcomes that may involve entering the feared situation but using subtle avoidance. Evidence from the systematic review includes reports of the hiding of bottle feeding and making excuses for bottle feeding [45]. Other examples of safety seeking behaviours might include only disclosing to other known HIV-positive people, attempting to assess the potential recipient’s HIV attitudes before deciding whether to disclose, or presenting a plausible alternative account for medication use [12]. Again, these strategies are not inherently unhelpful but they can become so if they play a part in a cycle that maintains perceived threat and this has a significant impact on functioning.

#### Cognitive Factors

Cognitive factors maintaining disclosure anxiety may include preoccupation with thoughts about disclosing or others finding out about one’s HIV status, negative rumination and self-focused attention in social situations where one’s HIV status is not known. There may be attentional biases, with a focus on attending to negative talk about people with HIV, as well as scanning for information about HIV and other people’s views about the condition. We suggest these factors based on the clinical anxiety literature [32, 33]. They have not been investigated, to our knowledge, in HIV-positive populations.

#### Affective and Physiological Factors

There is evidence that HIV disclosure concerns are positively associated with general levels of anxiety [20, 21]. High levels of general anxiety are likely to maintain beliefs about interpersonal threat relating to specific disclosure situations. We also propose that HIV disclosure anxiety is characterised by increased arousal (e.g., sweating, feeling hot) in situations where disclosure of one’s status is possible or required. This aversive nature of these symptoms and the desire to use avoidance to manage them serves to maintain anxiety, similar to processes occurring in social anxiety [32].

## Summary and Conclusions

We present a novel cognitive model of HIV disclosure anxiety that attempts to both explain the nature of high level of anxiety about sharing one’s HIV-positive status and to suggest avenues for therapeutic intervention. Our model is the first HIV disclosure model that we are aware of to focus on the role of anxiety and factors that maintain HIV disclosure threat responses. The model is based on robust clinical models of anxiety disorders, adapted for the specific context of HIV disclosure. Our model differs from health anxiety models [33] in having the interpretation of future interpersonal situations at its core versus the interpretation of one’s own symptoms. It also highlights a specific role for (a) deeper level cognitions about the self in relation to the condition (e.g., internalised HIV stigma beliefs) (b) context, compared with health anxiety models. Our model differs from social anxiety models [32] as it does not have self-focussed attention at its core, and instead highlights both a specific role for context, and provides more detail on the threat interpretation/feared prediction.

There is evidence for some aspects of the model (e.g., the relationship between HIV stigma and HIV disclosure anxiety, and catastrophic beliefs concerning HIV disclosure outcome). Many components of the model, however, remain to be investigated (and there is an absence of standardised measures of HIV disclosure anxiety or its determinants that will facilitate model testing). For example, there have not been any attempts to separate out the different elements of threat interpretation to investigate whether the model accurately describes and predicts disclosure anxiety. Although there are not current measures of these beliefs, they could be operationalised and measured reliably and validly in the future. Another limitation is that the model does not attempt to explain post-disclosure outcomes. Future research should attempt to operationalise key elements of the model (e.g., HIV disclosure anxiety, disclosure conditional assumptions, threat interpretations, and disclosure avoidance) so that relationships suggested by the model can be tested. In addition, the model would benefit from studies examining the prevalence of disclosure anxiety-related impact on quality of life and functioning in different HIV-positive populations.

Our model has clear clinical implications. In particular, we suggest that the existence of HIV disclosure anxiety should be normalised and the maintenance components of the model should be focused on in interventions where individuals are most affected by HIV disclosure anxiety and are motivated to consider sharing their status (threat interpretations, avoidance, safety-seeking behaviours, rumination, and attentional focus). Many of the cognitive-behavioural strategies used in treating health anxiety and social anxiety could be relevant for working with individuals who are most anxious about sharing their status. Such techniques could involve developing ways to challenge anxiogenic cognitions (e.g., cognitive restructuring and behavioural experiments), graded exposure to feared situations with the withdrawal of safety-seeking behaviours, teaching disclosure communication skills to enhance perceptions of coping, exploring different explanations for disclosure anxiety (Theory A versus Theory B), and accessing HIV disclosure support. In addition, motivational interviewing could be used to address disclosure ambivalence. The goals of such an intervention might be to help the individual to understand what maintains their high levels of anxiety, and to enable them to manage anxiety such that they can make a considered decision about how to respond to threat interpretations. This may help to break the HIV disclosure anxiety maintaining cycle, reducing both the preoccupation with HIV disclosure and disabling levels of anxiety. This may enable the sharing of one’s status *in situations where the individual wants to disclose,* or the management of anxiety in situations where one’s status is not known and there is no motivation to disclose.

We argue that developing a new theory-based intervention to minimise significant levels of HIV disclosure anxiety, is warranted, given the limited evidence of existing effective interventions to reduce anxiety about HIV disclosure from the systematic review [17–19]. It is interesting to note that the most promising of the interventions included in the review [17] used some of the components that we suggest might be beneficial.

## Electronic supplementary material

Below is the link to the electronic supplementary material.

Supplementary material 1 (DOCX 191 kb)
