# Supplementary material for: HIV Disclosure Anxiety: A Systematic Review and Theoretical Synthesis
Source: AIDS Behav. 2016 Jul 12;21(1):1–11. doi: 10.1007/s10461-016-1453-3 (PMC5216111; doi:10.1007/s10461-016-1453-3)
Supplement: Supplementary file 1 — Supplementary material 1 (DOCX 191 kb) [file 10461_2016_1453_MOESM1_ESM.docx]

**Table I: Disclosure anxiety studies**

| **Study** | **Location** | **Design (cross sectional unless stated)/**  **Methodology** | **Sample (all HIV+)** | **Disclosure recipient** | **Findings related to HIV disclosure anxiety** |
| --- | --- | --- | --- | --- | --- |
| **Akani & Erhabor (2006)**[**^1^**](#_ENREF_1) | Nigeria | Survey | 187 (82 females), 19-56 years. | Any | Fear of stigmatization, victimization, accusation of infidelity, and secondary disclosure cited as reasons for non-disclosure. |
| **Beauregard & Solomon (2005)**[**^2^**](#_ENREF_2) | Canada | Qualitative interviews | 5 females, 40-48 years. | Family | Fear of disclosure due to stigmatization and uncertainty as to how people would react. Fear of being abandoned by family members, particularly children, due to lack of understanding. |
| **Bhagwanjee et al (2011)**[**^3^**](#_ENREF_3) | South Africa | Qualitative interviews | 19 male mineworkers, 33-57 years. | Any | Fear of being abandoned, causing stress or being treated differently cited as reasons for not disclosing to partners and family. Stigmatisation stated as reason for not disclosing to non-family. |
| **Bohle, Dilger & Groß (2014)**[**^4^**](#_ENREF_4) | Tanzania | Participant observation, Survey. Qualitative interview | 59 females on ART (mean age 39 years). | Any | Fear of discrimination the main reason cited for non-disclosure. |
| **Brackis-Cott, Mellins & Block (2003)**[**^5^**](#_ENREF_5) | US | Focus groups | 12 HIV+ mothers, 30-48 years, (mean 40 years). | Children | All HIV+ mothers had disclosed to their adolescent children, despite anxiety. |
| **Braitstein et al (2011)**[**^6^**](#_ENREF_6) | Kenya | Survey | Parents of 97 children lost to follow up (45% of who were known to be HIV+). 43% female. Median age of child, 0.6 years. | Any | Most common reason given for not returning to the clinic was disclosure issues or fear of community or family discrimination (related to the mother’s own status or the child’s). |
| **Brickley et al (2009)**[**^7^**](#_ENREF_7) | Vietnam | Qualitative interviews and focus groups | Pregnant and postpartum females. 20 in interviews, 14 in focus groups.  19-34 years. | Any | Participants reported avoiding disclosure of their HIV status due to fear of stigma and discrimination, particularly in the wider community. |
| **Buchanan et al (2012)**[**^8^**](#_ENREF_8) | US | Survey | 120 perinatally infected children and adolescents (55 females), aged 8 to18 years, mean age 12.8 years, and their parents/ caregivers. | Any | Where the caregiver was fully responsible for adherence, there was significant agreement between caregiver and child on the reported barrier to ART adherence “child concerned that others notice medications” (k=0.41, p= .008). |
| **Busza et al (2013)**[**^9^**](#_ENREF_9) | Tanzania | Qualitative interviews | 14 perinatally infected (5 females), 15-19 years. | Partners | For those that acknowledged sexual feelings, concern about how to tell a potential partner about their status and how they might react. |
| **Bwirire et al (2008)**[**^10^**](#_ENREF_10) | Malawi | Focus groups | 10 antenatal and 6 postnatal females and 9 midwives, aged 20-55 years. | Partner (and community) | Fear of stigma, discrimination, household conflict and divorce on disclosing were reasons cited for loss to follow up in a PMTCT programme. |
| **Catz et al (2012)**[**^11^**](#_ENREF_11) | US | Qualitative interviews | 30 prisoners (9 females), 20-58 years (mean 39 years). | Partners | Fear of rejection cited as biggest deterrent to disclosing HIV status, and a barrier to risk reduction (e.g., condom use). |
| **Chakrapani et al (2010)**[**^12^**](#_ENREF_12) | India | Survey, focus groups and qualitative interviews | 200 in survey (100 males, mean 34 years; 100 females, mean 28.3 years); 58 in focus groups,  31 in Qualitative interviews. | Partners | Fear that disclosure will bring marital discord and family shame was a barrier to condom use. |
| **Chakrapani et al (2009)**[**^13^**](#_ENREF_13) | India | Focus groups | 19 female sex workers, 21-48 years (mean 33 years). | Any | Fear of adverse consequences after disclosure, in particular, stigma and discrimination by family and community, was cited as a barrier to seeking ART. |
| **Chandra et al (2003)**[**^14^**](#_ENREF_14) | India | Qualitative interviews | 68 (33 females), 18-50 years (mean 31 years). | Any (with emphasis on family) | Reasons given for non-disclosure included fear of discrimination. |
| **Chin & Kroesen (1999)**[**^15^**](#_ENREF_15) | US | Qualitative interviews | 9 Asian/Pacific Islander females, 21-51 years (mean 38.8 years). | Any | Disclosure decisions influenced by fears of being stigmatized, concerns about disappointing or burdening others and concerns about discrimination. |
| **Christianson, Lalos & Johansson (2008)**[**^16^**](#_ENREF_16) | Sweden | Qualitative interviews | 10 (5 females), 17-24 years. | Partners | One participant reported that fear of disclosure led them to ‘switch off’ lust, leading to a lower sex drive so that the need to disclose was avoided. |
| **Cloete et al (2010)**[**^17^**](#_ENREF_17) | South Africa | Focus groups | 83- Males and Females aged 18+  (Demographic info not collected) | Any | Fear of disclosure due to concerns about rejection by family or partners, and loss of job. |
| **Colombini et al (2014)**[**^18^**](#_ENREF_18) | Kenya | Qualitative interviews | 48 females (4 aged 18-24 years, 32 aged 25-34 years, 12 aged 35+ years). | Any | Fear of being discriminated against and being rejected cited as leading to non-disclosure, however this reportedly did not affect ART adherence and motivation to stay healthy. |
| **Conserve & King (2014)**[**^19^**](#_ENREF_19) | US | Qualitative interviews | 21 (16 females), 18+ years, of Haitian decent. | Any | Barriers to disclosure included the fear of being stigmatized and rejected, and also concern that it may cause confidants (usually close family or friends) to worry. |
| **Corona et al (2006)**[**^20^**](#_ENREF_20) | US | Survey | 274 parents (216 female) mean age 35.8 years. | Children | Reasons for non disclosure- worry about emotional consequences of disclosure (67%) and worry that child would tell other people (28%). |
| **Curioso et al (2010)**[**^21^**](#_ENREF_21) | Peru | Qualitative interviews | 31 on ART (3 females), 18+ years. | Any | Fear of disclosure was a barrier to ART adherence: participants discussed having to be secretive with their medication. |
| **Dafarty & Padayatchi (2012)**[**^22^**](#_ENREF_22) | US | Qualitative interviews | 40 with TB co-infection (24 females), 21-47 years (mean 34 years). | Any | Participants reported not adhering to ART due to fears of others seeing them taking the medication or going to collect it from the clinic. |
| **Dageid et al (2012)**[**^23^**](#_ENREF_23) | South Africa | Qualitative interviews and focus groups | 23 males in interviews, aged 22-73 years (median 39 years).  8 males in focus group, aged 22-47 years (median 32 years). | Any | Fear of rejection, isolation, tarnished reputations and perceived stigma given as reasons for non-disclosure. Five participants reported fearing violence following disclosure. Participants feared that loved ones may not be able to cope if they disclosed their HIV diagnosis to them. |
| **Degroote et al (2014)**[**^24^**](#_ENREF_24) | Belgium | Survey | 54 (4 females), 33-51 years (mean 42 years). | Workplace | Fear of social or professional consequences reported as a barrier to disclosure. |
| **Deribe et al (2008)**[**^25^**](#_ENREF_25) | Ethiopia | Survey | 705 (353 females) 166 aged 18-25 years, 358 aged 26-35 years, 181 aged >36 years. | Mainly partner. Also family, friends, church. | Fear of partner reacting negatively cited as reason for non-disclosure (e.g., anger. Rejection/separation, violence, accusations of infidelity, worry) |
| **Deribe et al (2009/2010)**[**^26^**](#_ENREF_26)**^,^**[**^27^**](#_ENREF_27) | Ethiopia | Survey and Qualitative interviews | 706 (353 males, mean 34 years; 353 females, mean 29 years). 11 in-depth interviews (4 males, 7 females). | Partner | Anxiety about worrying partner and revealing unfaithfulness were reasons for non-disclosure (men). Fear of violence, financial hardship, abandonment and being blamed cited as reasons for non-disclosure (women). |
| **Derlega et al (2002/ 2004)**[**^28^**](#_ENREF_28)**^,^**[**^29^**](#_ENREF_29) | US | Survey | 145 (39 females), Mean age 37 years. | Any | Perceived HIV-related stigma associated with reasons given for non-disclosure included fear of rejection. |
| **Diagne Gueye et al (2007)**[**^30^**](#_ENREF_30) | France | Survey | 54 mothers | Mainly partner, also family, friends, church | Fear of violence and separation were reasons for cited non-disclosure to partner. |
| **Dinkel (2014)**[**^31^**](#_ENREF_31) | Germany | Self-report questionnaire | 167 MSM, 22-74 years (mean 45 years) | Any | Disclosure concerns subscale of HIV Stigma Scale- associations with: HADS anxiety, r= 0.19, p<0.05; HADS depression, r= 0.14, ns; Life Satisfaction (general), r= -0.1, ns; Life Satisfaction (health), r= 0.01, ns; Perceived Social Support, r= -0.17, p<0.05. |
| **Doherty et al (2006)**[**^32^**](#_ENREF_32) | South Africa | Qualitative interviews | 40 mothers whom exclusively bottle/breast fed (mean 24 years). | Any | Some participants reported hiding the fact that they bottle feed their baby as they feared others would find out/ think that it was because they are HIV+. Participants also reported making up other excuses to avoid stigma e.g., the baby would not breastfeed/ having other illnesses. |
| **Donahue et al (2012)**[**^33^**](#_ENREF_33) | Malawi | Qualitative interviews | 59 females, 21-36 years. | Any | Fear of involuntary disclosure (and subsequent community stigmatisation) was a barrier to infant HIV testing and care programmes. |
| **Eisenhut et al (2009)**[**^34^**](#_ENREF_34) | UK | Survey | 62 mothers. | Any | A common reason given by mothers for not having their children tested was fear of disclosure to others (56%). |
| **Ekama et al (2012)**[**^35^**](#_ENREF_35) | Nigeria | Survey | 170 pregnant females 65 aged 18-29 years, 89 aged 30–34 years, 16 aged ≥ 35 years. | Any | Fear of being identified as HIV positive (63.6%) was the most common reason for nonadherence. |
| **Errol et al (2012)**[**^36^**](#_ENREF_36) | India | Qualitative interviews | 13 interviewed (8 males, 3 females, 2 transgender), aged 28-55 years. | Any | Participants reported fearing that neighbours or family would find out about their status or they would be forced to tell them due to home visits by staff from the HIV clinic. |
| **Ezechi et al (2009)**[**^37^**](#_ENREF_37) | Nigeria | Survey | 625 pregnant females.  270 aged 18-29 years,  322 aged 30-39 years,  33 aged 40+ years. | Partner | Fear of rejection, stigma, and possible abuse were cited as reasons for non-disclosure. |
| **Fesko (2001)**[**^38^**](#_ENREF_38) | US | Qualitative interviews | 18 (9 females), 2 aged 23-30 years, 9 aged 31-40 years, 7 aged 41-50 years. | Workplace | Fear of possible consequences such as being fired or rejected by co-workers were cited as reasons for non-disclosure. |
| **Fongkaew et al (2014)**[**^39^**](#_ENREF_39) | Thailand | Qualitative interviews | 30 (13 females), 14-21 years. | Any | Participants feared disclosure as they worried that they may be rejected by their partner or humiliated by their peer group due to stigmatisation and disgust; this reportedly led to non-adherence. |
| **Forsberg (1996)**[**^40^**](#_ENREF_40) | US | Survey | 306 with haemophilia co-infection, mean 19 years). | Partners | Participants feared that their partner would not want to have sex with them after disclosure or that their partner may tell someone else about their status. |
| **Gari, Habte & Markos (2010)**[**^41^**](#_ENREF_41) | Ethiopia | Survey | 384 females attending ART clinics, 18-57 years (mean 29.5 years). | Any | Fear of abandonment, break-up and stigma reported as barriers to disclosure. |
| **Gaskins (2006)**[**^42^**](#_ENREF_42) | US | Qualitative interviews | 20 African American males, 31-54 years. | Any | Concern about rejection and the disclosure recipients telling others were cited as barriers to disclosure. |
| **Gaskins et al (2011)**[**^43^**](#_ENREF_43) | US | Qualitative interviews | 40 African American males, 22-49 years (mean 38 years). | Any | Fear of negative reactions or stigma, and fear of the disclosure recipient telling others were common reasons reported for non-disclosure. |
| **Gielen et al (1997)**[**^44^**](#_ENREF_44) | US | Qualitative interviews | 50 females, 16-45 years. | Any | Reasons quoted for non-disclosure included concerns about rejection, discrimination and violence. |
| **Gillard & Roark (2013)**[**^45^**](#_ENREF_45) | US | Qualitative interviews and observations | 9 African American/ Hispanic 17-19 years. | Any | Participants reported amotivation to disclose due to fear of stigma and negative reactions/consequences, particularly if previous confidants had reacted negatively, e.g., gossiping and rejection. |
| **Gorbach et al (2004)**[**^46^**](#_ENREF_46) | US | Qualitative interviews | 55 MSM (mean 38.5 years). | Partners | Some men reported a fear of being rejected by a prospective  partner as a reason that they only disclosed sometimes. |
| **Gray (1999)**[**^47^**](#_ENREF_47) | US | Survey | 80 females, 20-65 years (mean 35.8 years). | Any | Fears related to disclosure and stigma reported more frequently than fears of dying. |
| **Greene et al (2013)**[**^48^**](#_ENREF_48) | US | Intervention study  Self-report questionnaire | 43 African American18 males, 25 females, 20-64 years (mean 47.3 years). | Any | Brief Disclosure Intervention (BDI). Disclosure anxiety measured by a single item asking participants to rate how anxious they feel before telling someone on a scale of 1-10. Participants also asked to describe how much they worry about telling others their status (rated from 0-5 by two coders reading the transcripts).  Controlling for length of time since diagnosis, there was a condition by time interaction (F (1, 21)= 20.54, p<.001, partial η2= .49) with a decrease in disclosure anxiety in the intervention group from Time 1 pretest to delayed posttest (pretest M= 4.41, SD= .89; delayed posttest M=3.76, SD= 1.22).  Controlling for length of time since diagnosis, there was a condition by time interaction (F(1, 40)= 3.70, p<.05, partial η2=.08) with a decrease in worry in the intervention group from pretest to immediate posttest (pretest M=3.68, SD=1.49; immediate posttest M=2.80, SD= 1.22) |
| **Grodensky et al (2015)**[**^49^**](#_ENREF_49) | US | Qualitative interviews | 15 females, 50+ years (mean 57 years). | Any | Fear of disclosure cited as a barrier to making church connections and reportedly led to isolation. |
| **Hardon et al (2013)**[**^50^**](#_ENREF_50) | Burkino Faso, Kenya, Malawi & Uganda | Survey | 157 (90 female), mean 34.5 years. | Partners | Fear of stigma, troubles being caused in the marriage and divorce cited as barriers to disclosure. |
| **Hays et al (1993)**[**^51^**](#_ENREF_51) | US | Survey | 165 MSM, 24-68 years (mean 39.6 years). | Any | Fear of discrimination or disruptions of relationships were given as reasons for non-disclosure. |
| **Hogwood, Campbell & Butler (2013)**[**^52^**](#_ENREF_52) | UK | Qualitative interviews | 9 (7 females), 13-19 years (mean 16 years). | Any | Participants reported non-disclosure due to fear of rejection and isolation, and also a fear of losing control of the information due to others not maintaining secrecy. |
| **Holmes & Shea (1997)**[**^53^**](#_ENREF_53) | US | Self-report questionnaire | 106 asymptomatic (28 females), mean 37.8 years. | Any | More disclosure worries (as measured by HAT QoL) in heterosexual versus homosexual / bisexual (p<0.05) and in females than males (p<0.05). |
| **Holmes & Shea (1998)**[**^54^**](#_ENREF_54) | US | Self-report questionnaire | 201 (44 females), mean 37.5 years. | Any | More disclosure worries (as measured by HAT QoL) associated with higher CD4 count (p<0.05), non-AIDS diagnosis (versus AIDS)(p<0.01), no HIV-related hospitalization (p<0.01), younger age (p<0.05), heterosexual versus gay/bisexual (p<0.01), no partner versus partner (p<0.05). |
| **Holmes & Shea (1999)**[**^55^**](#_ENREF_55) | US | Self-report questionnaire | 215 (42 females), mean 37.8 years. | Any | More disclosure worries (as measured by HAT QoL) reported in heterosexuals and those with non-AIDS diagnosis (vs. AIDS), p<0.01. |
| **Hsuing & Tsai (2000)**[**^56^**](#_ENREF_56) | Taiwan | Qualitative interviews | 14 males (mean 36 years). | Any | Fear of disclosure was a stressor. |
| **Issiaka et al (2001)**[**^57^**](#_ENREF_57) | Burkina Faso | Survey | 79 females, 18-38 years (mean 24.5 years). | Partner | Most frequently cited reasons for not disclosing to partner were fears of rejection/abandonment and fears of being considered unfaithful. |
| **Johnson (2012)**[**^58^**](#_ENREF_58) | Nigeria | Survey | 331 (164 females), 31 aged 15-24 years, 150 aged 25-34 years, 150 aged 35> years. | Any | Fear of stigmatization resulted in low disclosure outside immediate family (e.g., to colleagues or community). |
| **Kadowa & Nuwaha (2009)**[**^59^**](#_ENREF_59) | Uganda | Survey | 278 (197 females). 139 had not disclosed- Mean age 31 years. | Any | Fear of divorce or violence, discrimination or stigma, rumours/ gossip, and accusations of promiscuity/infidelity cited as reasons for non-disclosure. |
| **Kerrigan (2006)**[**^60^**](#_ENREF_60) | Brazil | Qualitative interviews | 10 heterosexual females (mean 37 years), 10 heterosexual males (mean 38.5 years) and 10 MSM (Mean 40 years). | Partners | Women: fears related to disclosure were centred on the potential for negative partner reactions including rejection, abandonment and violence;  Heterosexual men: fear of being labelled/ being treated differently;  MSM: Fear of how partner would respond, risk of getting killed mentioned with casual partners. |
| **Kilewo et al (2001)**[**^61^**](#_ENREF_61) | Tanzania | Survey | 288 pregnant females, 18-43 years (mean 27.7 years). | Partner | Fear of stigma, divorce and violence were reasons given for non-disclosure to partners. |
| **Klopper et al (2014)**[**^62^**](#_ENREF_62) | South Africa | Survey | 150 (106 females) Mean 36 years. | Any | Fear of stigmatization, especially among males, cited as a major reason for delayed/non-disclosure.  Fear of stigmatization appeared to be associated with higher level of education. Fear of blame/discrimination appeared to be associated with less disclosure to sexual partner; more fear if no personal income and not married to partner. |
| **Kumar et al (2006)**[**^63^**](#_ENREF_63) | Barbados | Qualitative Interviews | 139 females. 72 aged <25 years, 67 >25 years. | Any | Fear of stigmatization by anyone, and fear of abnormal reaction and possible violence from partner cited as reasons for non-disclosure. |
| **Kyaddondo et al (2013)**[**^64^**](#_ENREF_64) | Uganda | Focus groups and survey | Focus groups – 16 parents (8 females).  Survey - 148 parents (100 females). 32 aged 18- 24 years; 59 aged 25- 34 years; 57 35+ years. | Children | Reasons for non-disclosure to children include fear of being blamed/ judged as being sexually irresponsible, fears of rejection/isolation. |
| **Laryea & Gien (1993)**[**^65^**](#_ENREF_65) | Canada | Qualitative interviews | 25 (6 females), 18-42 years. | Any | Fears of rejection and discrimination were a barrier to disclosure. Fear of secret being shared, and the concern that this may prevent them from getting a job, particularly in small communities. |
| **Lee et al (2013)**[**^66^**](#_ENREF_66) | Thailand | Focus groups and qualitative interviews | Focus groups - 40; Interviews- 50 (35 female), mean age 37.5 years. | Any | Fear of rejection and disapproval, fear of breaches of privacy, perceived stigma and shame were barriers to disclosure. |
| **Liamputtong et al (2014)**[**^67^**](#_ENREF_67) | Thailand | Qualitative interviews | 26 females. 4 20-30 years, 17 31-40 years, 5 >40 years. | Family and children | Non-disclosure due to fear of stigma, discrimination, blame and rejection. Worries about their partners’ well-being, as it would be assumed that they had HIV too. Concern that family members/children would not cope with disclosure. |
| **Lugalla et al (2012)**[**^68^**](#_ENREF_68) | Tanzania | Qualitative interviews | 57 (27 females), most 20-39 years. | Any | Fear of being rejected and discriminated against were main reasons for non-disclosure in general. Both sexes reported finding it particularly difficult/ were very reluctant to disclose to their fathers. Women less likely to disclose to partners than men due to fear of violence and abandonment. |
| **Madiba & Canti-Sigaqa (2012)**[**^69^**](#_ENREF_69) | South Africa | Focus groups | 50 men aged 28-70 | Any | Several people reported that they did not want to attend support groups as they were afraid of exposing themselves and everyone knowing they were HIV+.  One participant stated that the other participants in support groups are not trustworthy so he would not want to disclose his status there. |
| **Madiba (2013)**[**^70^**](#_ENREF_70) | South Africa | Focus groups | 26 parents (18 females), 20-60 years. | Children | Fear of disclosure reported due to fear of onward disclosure from child, fear that the child is not mature enough to cope, and anticipated stigma and discrimination. |
| **Maiorana et al (2012)**[**^71^**](#_ENREF_71) | US | Qualitative interviews | 52 who had participated in interventions purposively selected to represent clinics’ demographics. | Any | Positive prevention interventions reported to help explore fears of stigma and rejection associated with disclosure. |
| **Maliska et al (2009)**[**^72^**](#_ENREF_72) | Brazil | Qualitative interviews | 13 | Family and friends | Fear of disclosure associated with stigma. |
| **Maman et al (2001)**[**^73^**](#_ENREF_73) | Tanzania | Qualitative interviews | 62- 26 female, 36 males. Mean age 28 years. (Only 27 HIV+). | Partners | Women were more anxious about disclosing their positive test results than men, reportedly worrying about blame, abandonment and abuse. HIV+ men reported less disclosure anxiety, describing it as ‘like a normal conversation’. |
| **Maman et al (2003)**[**^74^**](#_ENREF_74) | Tanzania | Survey | 245 females, 3 months after testing. Mean age 32 years. (Only 73 HIV+). | Partners | Fear of partner’s reaction was the main reason cited for non-disclosure. Particular fear of abandonment and loss of economic support. |
| **Manopaiboon et al (1998)**[**^75^**](#_ENREF_75) | Thailand | Survey | 129 pregnant females,15-37 years (median 22 years). | Friends and family | Non-disclosure associated with fear of disclosure outcome (e.g., family feeling ashamed). |
| **Mayanja et al (2013)**[**^76^**](#_ENREF_76) | Uganda | Survey | 421 pre-ART (271 females). 318 > 30 years | Any | Fear of disclosure was most common worry before starting antiretroviral therapy. |
| **Mburu et al (2014)**[**^77^**](#_ENREF_77) | Zambia | Qualitative interviews and focus groups | Focus groups- 53; Interviews- 58 (29 females), mean 16.8 years. | Partners | Fear of abandonment/ rejection (particularly by partners) and stigma were barriers to disclosure. |
| **Musumari et al (2013)**[**^78^**](#_ENREF_78) | DRC | Qualitative interviews | 38 (24 females), median age 41 years. | Any | Fear of rejection and gossip cited as reasons for non-disclosure. Participants reported interrupting medication out of fear of disclosing status. |
| **Nagikosi et al (2013)**[**^79^**](#_ENREF_79) | Uganda | Qualitative interviews | 48 refusing to engage in HIV care. Aged 15-49 years. | Any | Fear of stigma, rejection, blame, exclusion and devaluation were reported as most common barriers to entering HIV care. Some also feared losing social support from caretakers if status was disclosed, or feared that it would prevent them from carrying out their own caring obligations for family. |
| **Nam et al (2009)**[**^80^**](#_ENREF_80) | Botswana | Qualitative interviews | 21 parents (12 females), 22-55 years (mean 37 years). | Children | Concern that others may find out about their status and fear of children being stigmatized were reasons cited for non-disclosure. |
| **Newman et al (2007)**[**^81^**](#_ENREF_81) | Australia | Qualitative interviews | 20 (16 females), 22-54 years. | Any | Fear of disclosure and discrimination due to shame associated with HIV were major barriers to treatment uptake. |
| **Okoronkwo et al (2013)**[**^82^**](#_ENREF_82) | Nigeria | Survey | 221 attending ART clinic (126 females). 59 20-29 years, 74 30-39 years, 55 40-49 years. | Any | 188 admitted non-adherence. Fear of partner disclosure was a barrier to adherence in 12.7% of females and 21% of males. |
| **Olagbuji et al (2011)**[**^83^**](#_ENREF_83) | Nigeria | Survey | 166 pregnant females on ART , 25-39 years (mean 31.6 years) | Partner | Fear of spread of information, stigmatization and deterioration in relationships were most common reasons for non-disclosure. |
| **Osinde, Kakaire & Kaye (2012)**[**^84^**](#_ENREF_84) | Uganda | Survey | 403 (74% female, 83% on ART).  27.1% 25-29 years. | Partners | Fear of stigma was main reason for non-disclosure. Some participants also reported fearing abuse, abandonment and rejection from partners if they disclosed to them. |
| **Paiva et al (2011)**[**^85^**](#_ENREF_85) | Brazil | Qualitative interviews | 21 adolescents (10 female). 13-20 years. | Partners | Fear of how their partner would react and fear of ending up alone were reasons cited for not disclosing HIV status to partner. |
| **Patel et al (2012)**[**^86^**](#_ENREF_86) | India | Qualitative interviews | 30 (15 females), 15 aged 20-30 years, 9 aged 31-40 years, 6 aged 41-60 years. | Any | Fear of discrimination and fear of family breakdown were cited as barriers to disclosure. |
| **Petrak et al (2001)**[**^87^**](#_ENREF_87) | UK | Survey | 95 (79 males, mean 37.7 years;  16 females, mean 32.6 years | Any | Most frequently cited reasons for non-disclosure included protecting themselves from others’ negative reactions, discrimination, stigma and concerns about confidentiality. |
| **Phaladze et al (2005)**[**^88^**](#_ENREF_88) | Botswana, Lesotho, South Africa, Swaziland | Survey | 743 (453 females) Mean age 34 years. | Any | Disclosure worries (as measured by the HAT-QoL) independently associated with reduced life satisfaction (p< .001). |
| **Pugatch et al (2002)**[**^89^**](#_ENREF_89) | US | Semi-structured interviews | 6 (3 females). Aged 16-24 years. | Family | Fear of social stigma relating to HV disclosure was a barrier to ART adherence. |
| **Rosen & Ketlapile (2010)**[**^90^**](#_ENREF_90) | South Africa | Survey | 260 lost to follow-up. | Any | Fear of disclosure cited as a reason for loss to follow-up. |
| **Rothberg & Van Huyssteen (2008)**[**^91^**](#_ENREF_91) | South Africa | Survey | 28 (21 males, mean 40.5 years;  7 females, mean 38.6 years) | Workplace | Fear of disclosure of HIV status and stigmatisation were reasons cited for late registration onto a HIV treatment programme/ not using any support programmes. Participants also reported fear that employer will not maintain confidentiality. |
| **Rujumba et al (2012)**[**^92^**](#_ENREF_92) | Uganda | Qualitative interviews | 15 pregnant females, aged18-43 years. | Partners | Non-disclosure due to fear of abandonment, violence and accusation of bringing HIV into the family. |
| **Schlebusch & Vawda (2010)**[**^93^**](#_ENREF_93) | South Africa | Survey | 112 who had attempted suicide (82 females, 30 males), 18-48 years (mean 34.9 years). | Any | 54% of participants reported fear of disclosure of their HIV status because of possible victimisation/stigmatisation. |
| **Seid, Wasie & Admassu (2012)**[**^94^**](#_ENREF_94) | Ethiopia | Survey | 360 (191 females), mean 33.4 years. | Partners | Fear of divorce, stigma and discrimination, and physical abuse were main reasons given for non-disclosure. |
| **Sethosa & Peltzer (2005)**[**^95^**](#_ENREF_95) | South Africa | Qualitative interviews | 55 (41 females) recently diagnosed (mean 27.9 years). | Any | Reasons for non-disclosure included fear of negative reactions, discrimination, violence, and concerns about confidentiality. |
| **Siegel, Lekas & Schrimshaw (2005)**[**^96^**](#_ENREF_96) | US | Qualitative interviews | 158 females, mean 36 years. | Partners | Both pre and post ART periods characterized by fears that disclosure would be met with rejection or a breach of confidentiality. |
| **Siu et al (2012)**[**^97^**](#_ENREF_97) | Uganda | Focus groups and qualitative interviews | 20 (10 females),15-23 years (Median 19 years) | Any | Fear of secondary disclosure, discrimination, rejection and abuse cited as barriers to disclosure. |
| **Smith & Rapkin (1996)**[**^98^**](#_ENREF_98) | US | Qualitative interviews | 224 (30 females). 73 aged 24-35 years, 102 aged 36-45 years,  49 aged 46-56 years. | Any | Fear of disclosure of AIDS cited as a barrier to getting support from friends and family. |
| **Ssali et al (2010)**[**^99^**](#_ENREF_99) | Uganda | Qualitative interviews | 40 (20 females). 10 of each sex > 35 years, 10 of each sex < 35 years old. | Any | Fear of abandonment, particularly in young women disclosing to their partner, a common reason cited for non-disclosure. Fear of upsetting/worrying the disclosure recipient, particularly family members, given as another reason for non-disclosure. |
| **Stinson & Myer (2012)**[**^100^**](#_ENREF_100) | South Africa | Survey | 28 females (17 pregnant, 11 postpartum), mean 27 years. | Any | Fear of disclosing HIV status was reported as a barrier for initiating ART during pregnancy. Fear of the confidant having a negative reaction (e.g., abandonment or stigmatisation). |
| **Stutterheim et al (2011)**[**^101^**](#_ENREF_101) | Netherlands | Qualitative interviews | 42 of African and Afro-Caribbean descent, aged 18-70 years. | Any | Fear of stigmatization was most prevalent reason stated for non-disclosure. Sparing others, particularly parents and children, from worry another reason cited for non-disclosure. |
| **Syed et al (2014)**[**^102^**](#_ENREF_102) | Malaysia | Qualitative interviews | 13 (1 female), mean 34.4 years. | Any | Main reason given for non-disclosure was that they did not want to hurt their family’s emotions. Fear of stigma and discrimination was also reportedly linked to non-disclosure. |
| **Terry, Jones & Brown (1994)**[**^103^**](#_ENREF_103) | New Zealand | Survey | 57 (4 females).  12 aged 18-29 years, 21 aged 30-39 years, 24 aged 40-59 years. | Dentist | Reasons given by participants for not disclosing their HIV status to their dentist included fear of rejection, discrimination or breach of confidentiality. |
| **Teti et al (2010)**[**^104^**](#_ENREF_104) | US | Survey, Qualitative interviews | Survey (184 females)  Qualitative (18 females), 34- 51 years. | Any | Fear of rejection and retaliation cited as reasons to be cautious about disclosing. |
| **Thanh, Moland & Fylkesnes (2009)**[**^105^**](#_ENREF_105) | Vietnam | Qualitative interviews, focus groups, participant observation | 45 substance users (5 females), 21-45 years. | Any | Fear of rejection and loss of intimacy reportedly made disclosure difficult and was said to be an obstacle to condom use in those recently diagnosed. |
| **Thomas, Nyamathi &**  **Swaminathan (2009)**[**^106^**](#_ENREF_106) | India | Focus groups | 60 mothers, 23- 42 years (mean 30 years). | Children | Mothers reported that they feared disclosure may cause their children to discriminate against them, do badly in school, or tell others. |
| **Titilope et al (2011)**[**^107^**](#_ENREF_107) | Nigeria | Uncontrolled intervention  Survey | 499 (342 females), mean 37.3 years. | Partners | Fear of rejection was most common reason for non-disclosure. Of those who had not yet disclosed their status, 30.7% reported that counselling had helped them overcome the fear of not wanting to disclose, but only 18.8% stated that they were willing to disclose. |
| **Tzemis et al (2013)**[**^108^**](#_ENREF_108) | Canada | Self-report questionnaire | 775 on ART (25% females), 40-52 years (median 45 years). | Any | Disclosure concerns associated with HIV related stigma in both univariable and multivariable analysis (< .001). |
| **Unge et al (2008)**[**^109^**](#_ENREF_109) | Kenya | Qualitative interviews | 26 (17 females), 23-55 years. | Any | Fear of disclosure and its negative repercussions associated with not initiating ART. |
| **Vallerand et al (2005)**[**^110^**](#_ENREF_110) | US | Qualitative interviews | 35 females, aged 27-56 years (mean 38.5 years). | Children | Mothers expressed fears of rejection by their child or other potential negative reactions from their children if they disclosed to them. |
| **Van Devanter et al (2011)**[**^111^**](#_ENREF_111) | US | Qualitative interviews | 26 Black and Latina females, 16-24 years | Partners | Fear of disclosure to new partners associated with risky sex (not using a condom). |
| **Van Nuil et al (2014)**[**^112^**](#_ENREF_112) | Rwanda | Focus groups | 42 on ART , aged 12-21 years (median 17 years). | Partner | One of the most commonly reported HIV-related anxieties was how and when to disclose to a partner. |
| **Vance & Woodley (2005)**[**^113^**](#_ENREF_113) | US | Qualitative interviews | 12 (2 females), 38-50 years, (mean 44.4 years). | Any | Perceived barriers to successful ageing included fear of disclosure and stigma. |
| **Varni et al (2012)**[**^114^**](#_ENREF_114) | US | Self-report questionnaire | 200 (72% male) aged 18-64 years (Mean age 43.17 years old). | Any | The disclosure concerns measure had a significant positive correlation with enacted stigma (r= .27, p≤ .01), concern with public attitudes (r= .47, p≤ .01), negative self-image (r= .46, p≤ .01), disengagement coping (r= .37, p≤ .01), depression (r= .24, p≤ .01), and anxiety (r= .21, p≤ .01).  The disclosure concerns measure had a significant negative correlation with time (years) since diagnosis (r= -.14, p≤ .05), primary control engagement coping (r= -.26, ≤ .01), and self-esteem (r= -.18, p≤ .05). |
| **Visser et al (2008)**[**^115^**](#_ENREF_115) | South Africa | Survey | 293 recently diagnosed pregnant females (mean 26.5 years). | Any | Fear of abandonment and discrimination weighed against need for support and desire to raise risk awareness in disclosure decisions. |
| **Walker (2012)**[**^116^**](#_ENREF_116) | US | Qualitative interviews | 13 mothers. | Any | Many women reported living in isolation and fearing disclosure. |
| **Ware, Wyatt & Tugenberg (2006)**[**^117^**](#_ENREF_117) | US | Qualitative interviews | 52 (38 male, 14 female), 28 30-40 years, 24 41-51 years. | Any | Fear of disclosure was reported to result in missing medication as the interviewees did not want others to see them taking pills. |
| **Webel (2010)**[**^118^**](#_ENREF_118) | US | Intervention  Self-report questionnaire | 89 females (mean 47 years). | Any | Peer based HIV symptom management intervention consisting of 7 two hour sessions, with the content of the sessions based on the Positive Self-Management Program (PSMP). The control group received a symptom management guide book.  There was a significant difference found between groups for disclosure worries as measured by the HAT-QoL (χ2= 24.67, p<0.005). However, inspection of the means indicate that there was no significant group x time interaction. |
| **Wolf et al (2014)**[**^119^**](#_ENREF_119) | Kenya | Qualitative interviews, focus groups | 27 (19 females), 15-21 years. | Adults | Fear of status being disclosed to family reportedly affected medication adherence and caused loss-to-follow up. Also, youths’ dependent relationships with adults at home and school were reportedly negatively impacted by youths’ fear of disclosure caused by HIV-related stigma. |
| **Zhou et al (2013)**[**^120^**](#_ENREF_120) | China | Qualitative interviews | 39 parents (13 females), 28-76 years (mean 42 years). | Children | Fear of being stigmatized, fear of rejection, and fear of increased psychological burden to children were reasons for non-disclosure. |
| **Zukoski, Thorburn & Stroud (2011)**[**^121^**](#_ENREF_121) | US | Qualitative interviews | 16 (7 female). 3 18-34 years, 6 35-49 years, 7 50+ years. | Any, particularly health profs. | Fear of disclosure cited as a barrier to seeking information about HIV. |

1. Akani CI, Erhabor O. Rate, pattern and barriers of HIV serostatus disclosure in a resource-limited setting in the Niger delta of Nigeria. *Tropical doctor.* Apr 2006;36(2):87-89.

2. Beauregard C, Solomon P. Understanding the experience of HIV/AIDS for women: implications for occupational therapists. *Canadian journal of occupational therapy. Revue canadienne d'ergotherapie.* Apr 2005;72(2):113-120.

3. Bhagwanjee A, Govender K, Akintola O, et al. Patterns of disclosure and antiretroviral treatment adherence in a South African mining workplace programme and implications for HIV prevention. *African journal of AIDS research : AJAR.* 2011;10 Suppl 1:357-368.

4. Bohle LF, Dilger H, Gross U. HIV-serostatus disclosure in the context of free antiretroviral therapy and socio-economic dependency: experiences among women living with HIV in Tanzania. *African journal of AIDS research : AJAR.* Sep 2014;13(3):215-227.

5. Brackis-Cott E, Mellins CA, Abrams E, Reval T, Dolezal C. Pediatric HIV medication adherence: the views of medical providers from two primary care programs. *Journal of pediatric health care : official publication of National Association of Pediatric Nurse Associates & Practitioners.* Sep-Oct 2003;17(5):252-260.

6. Braitstein P, Songok J, Vreeman RC, et al. "Wamepotea" (they have become lost): outcomes of HIV-positive and HIV-exposed children lost to follow-up from a large HIV treatment program in western Kenya. *Journal of acquired immune deficiency syndromes.* Jul 1 2011;57(3):e40-46.

7. Brickley DB, Le Dung Hanh D, Nguyet LT, Mandel JS, Giang le T, Sohn AH. Community, family, and partner-related stigma experienced by pregnant and postpartum women with HIV in Ho Chi Minh City, Vietnam. *AIDS and behavior.* Dec 2009;13(6):1197-1204.

8. Buchanan AL, Montepiedra G, Sirois PA, et al. Barriers to medication adherence in HIV-infected children and youth based on self- and caregiver report. *Pediatrics.* May 2012;129(5):e1244-1251.

9. Busza J, Besana GV, Mapunda P, Oliveras E. "I have grown up controlling myself a lot." Fear and misconceptions about sex among adolescents vertically-infected with HIV in Tanzania. *Reproductive health matters.* May 2013;21(41):87-96.

10. Bwirire LD, Fitzgerald M, Zachariah R, et al. Reasons for loss to follow-up among mothers registered in a prevention-of-mother-to-child transmission program in rural Malawi. *Transactions of the Royal Society of Tropical Medicine and Hygiene.* Dec 2008;102(12):1195-1200.

11. Catz SL, Thibodeau L, BlueSpruce J, et al. Prevention needs of HIV-positive men and women awaiting release from prison. *AIDS and behavior.* Jan 2012;16(1):108-120.

12. Chakrapani V, Newman PA, Shunmugam M, Dubrow R. Prevalence and contexts of inconsistent condom use among heterosexual men and women living with HIV in India: implications for prevention. *AIDS patient care and STDs.* Jan 2010;24(1):49-58.

13. Chakrapani V, Newman PA, Shunmugam M, Kurian AK, Dubrow R. Barriers to free antiretroviral treatment access for female sex workers in Chennai, India. *AIDS patient care and STDs.* Nov 2009;23(11):973-980.

14. Chandra PS, Deepthivarma S, Manjula V. Disclosure of HIV infection in south India: patterns, reasons and reactions. *AIDS care.* Apr 2003;15(2):207-215.

15. Chin D, Kroesen K. Disclosure of HIV infection among Asian/Pacific Islander American women: Cultural stigma and support. . *Cultural Diversity and Ethnic Minority Psychology.* 1999;5(3):222-235.

16. Christianson C, Lalos A, Johansson E. The Law of Communicable Diseases Act and disclosure to sexual partners among HIV-positive youth. *Vulnerable Children and Youth Studies.* 2008;3(3):234-242.

17. Cloete A, Strebel A, Simbayi L, van Wyk B, Henda N, Nqeketo A. Challenges Faced by People Living with HIV/AIDS in Cape Town, South Africa: Issues for Group Risk Reduction Interventions. *AIDS research and treatment.* 2010;2010:420270.

18. Colombini M, Mutemwa R, Kivunaga J, Stackpool Moore L, Mayhew SH, Integra I. Experiences of stigma among women living with HIV attending sexual and reproductive health services in Kenya: a qualitative study. *BMC health services research.* 2014;14:412.

19. Conserve DF, King G. An examination of the HIV serostatus disclosure process among Haitian immigrants in New York City. *AIDS care.* 2014;26(10):1270-1274.

20. Corona R, Beckett MK, Cowgill BO, et al. Do children know their parent's HIV status? Parental reports of child awareness in a nationally representative sample. *Ambulatory pediatrics : the official journal of the Ambulatory Pediatric Association.* May-Jun 2006;6(3):138-144.

21. Curioso WH, Kepka D, Cabello R, Segura P, Kurth AE. Understanding the facilitators and barriers of antiretroviral adherence in Peru: a qualitative study. *BMC public health.* 2010;10:13.

22. Daftary A, Padayatchi N. Social constraints to TB/HIV healthcare: accounts from coinfected patients in South Africa. *AIDS care.* 2012;24(12):1480-1486.

23. Dageid W, Govender K, Gordon SF. Masculinity and HIV disclosure among heterosexual South African men: implications for HIV/AIDS intervention. *Culture, health & sexuality.* 2012;14(8):925-940.

24. Degroote S, Vogelaers D, Koeck R, Borms R, De Meulemeester L, Vandijck D. HIV disclosure in the workplace. *Acta clinica Belgica.* Jun 2014;69(3):191-193.

25. Deribe K, Woldemichael K, Wondafrash M, Haile A, Amberbir A. Disclosure experience and associated factors among HIV positive men and women clinical service users in Southwest Ethiopia. *BMC public health.* 2008;8:81.

26. Deribe K, Woldemichael K, Bernard N, Yakob B. Gender difference in HIV status disclosure among HIV positive service users. *East African journal of public health.* Dec 2009;6(3):248-255.

27. Deribe K, Woldemichael K, Njau BJ, Yakob B, Biadgilign S, Amberbir A. Gender differences regarding barriers and motivators of HIV status disclosure among HIV-positive service users. *SAHARA J : journal of Social Aspects of HIV/AIDS Research Alliance / SAHARA , Human Sciences Research Council.* Jul 2010;7(1):30-39.

28. Derlega VJ, Winstead BA, Greene K, Serovich J, Elwood WN. Perceived HIV-related Stigma and HIV Disclosure to Relationship Partners after Finding Out about the Seropositive Diagnosis. *Journal of health psychology.* Jul 2002;7(4):415-432.

29. Derlega V, Winstead BA, Greene K, Serovich J, Elwood WN. Reasons for HIV Disclosure/Nondisclosure in Close Relationships: Testing a Model of HIV–Disclosure Decision Making. *Journal of social and clinical psychology.* 2004;23(6):747-767.

30. Diagne Gueye N, Dollfus C, Tabone M, et al. Psychosocial issues in HIV positive women during the periniatal period. *Archives de pediatrie.* 2007;14:461-466.

31. Dinkel A, Nather C, Jaeger H, et al. [Stigmatization in HIV/AIDS: first German adaptation of the HIV-stigma scale (HSS-D)]. *Psychotherapie, Psychosomatik, medizinische Psychologie.* Jan 2014;64(1):20-27.

32. Doherty T, Chopra M, Nkonki L, Jackson D, Greiner T. Effect of the HIV epidemic on infant feeding in South Africa: "When they see me coming with the tins they laugh at me". *Bulletin of the World Health Organization.* Feb 2006;84(2):90-96.

33. Donahue MC, Dube Q, Dow A, Umar E, Van Rie A. "They have already thrown away their chicken": barriers affecting participation by HIV-infected women in care and treatment programs for their infants in Blantyre, Malawi. *AIDS care.* 2012;24(10):1233-1239.

34. Eisenhut M, Kawsar M, Connan M, Balachandran T. Why are HIV-positive mothers refusing to have their children screened for vertically transmitted HIV infection? *International journal of STD & AIDS.* Jul 2009;20(7):506-507.

35. Ekama SO, Herbertson EC, Addeh EJ, et al. Pattern and determinants of antiretroviral drug adherence among Nigerian pregnant women. *Journal of pregnancy.* 2012;2012:851810.

36. Errol L, Isaakidis P, Zachariah R, et al. Tracing patients on antiretroviral treatment lost-to-follow-up in an urban slum in India. *Journal of advanced nursing.* Nov 2012;68(11):2399-2409.

37. Ezechi OC, Gab-Okafor C, Onwujekwe DI, Adu RA, Amadi E, Herbertson E. Intimate partner violence and correlates in pregnant HIV positive Nigerians. *Archives of gynecology and obstetrics.* Nov 2009;280(5):745-752.

38. Fesko SL. Disclosure of HIV status in the workplace: considerations and strategies. *Health & social work.* Nov 2001;26(4):235-244.

39. Fongkaew W, Viseskul N, Suksatit B, et al. Verifying quantitative stigma and medication adherence scales using qualitative methods among Thai youth living with HIV/AIDS. *Journal of the International Association of Providers of AIDS Care.* Jan-Feb 2014;13(1):69-77.

40. Forsberg AD, King G, Delaronde SR, Geary MK. Maintaining safer sex behaviours in HIV-infected adolescents with haemophilia. The Hemophilia Behavioral Evaluative Intervention Project Committee. *AIDS care.* Dec 1996;8(6):629-640.

41. Gari T, Habte D, Markos E. HIV positive status disclosure among women attending art clinic at Hawassa University Referral Hospital, South Ethiopia. *East African journal of public health.* Mar 2010;7(1):87-91.

42. Gaskins SW. Disclosure decisions of rural African American men living with HIV disease. *The Journal of the Association of Nurses in AIDS Care : JANAC.* Nov-Dec 2006;17(6):38-46.

43. Gaskins S, Payne Foster P, Sowell R, Lewis T, Gardner A, Parton J. Reasons for HIV disclosure and non-disclosure: an exploratory study of rural African American men. *Issues in mental health nursing.* 2011;32(6):367-373.

44. Gielen AC, O'Campo P, Faden RR, Eke A. Women's disclosure of HIV status: experiences of mistreatment and violence in an urban setting. *Women & health.* 1997;25(3):19-31.

45. Gillard A, Roark B. Older adolescents' self-determined motivations to disclose their HIV status. *Journal of Child Family Studies.* 2013;22:672-683.

46. Gorbach PM, Galea JT, Amani B, et al. Don't ask, don't tell: patterns of HIV disclosure among HIV positive men who have sex with men with recent STI practising high risk behaviour in Los Angeles and Seattle. *Sexually transmitted infections.* Dec 2004;80(6):512-517.

47. Gray JJ. The difficulties of women living with HIV infection. *Journal of psychosocial nursing and mental health services.* May 1999;37(5):39-43.

48. Greene K, Carpenter A, Catona D, Magsamen-Conrad K. The Brief Disclosure Intervention (BDI): Facilitating African Americans' Disclosure of HIV. *J Commun.* Feb 2013;63(1):138-158.

49. Grodensky CA, Golin CE, Jones C, et al. "I should know better": the roles of relationships, spirituality, disclosure, stigma, and shame for older women living with HIV seeking support in the South. *The Journal of the Association of Nurses in AIDS Care : JANAC.* Jan-Feb 2015;26(1):12-23.

50. Hardon A, Gomez GB, Vernooij E, et al. Do support groups members disclose less to their partners? The dynamics of HIV disclosure in four African countries. *BMC public health.* 2013;13:589.

51. Hays RB, McKusick L, Pollack L, Hilliard R, Hoff C, Coates TJ. Disclosing HIV seropositivity to significant others. *Aids.* Mar 1993;7(3):425-431.

52. Hogwood J, Campbell T, Butler S. I wish I could tell you but I can't: adolescents with perinatally acquired HIV and their dilemmas around self-disclosure. *Clinical child psychology and psychiatry.* Jan 2013;18(1):44-60.

53. Holmes WC, Shea JA. Performance of a new, HIV/AIDS-targeted quality of life (HAT-QoL) instrument in asymptomatic seropositive individuals. *Quality of life research : an international journal of quality of life aspects of treatment, care and rehabilitation.* Aug 1997;6(6):561-571.

54. Holmes WC, Shea JA. A new HIV/AIDS-targeted quality of life (HAT-QoL) instrument: development, reliability, and validity. *Medical care.* Feb 1998;36(2):138-154.

55. Holmes WC, Shea JA. Two approaches to measuring quality of life in the HIV/AIDS population: HAT-QoL and MOS-HIV. *Quality of life research : an international journal of quality of life aspects of treatment, care and rehabilitation.* Sep 1999;8(6):515-527.

56. Hsuing P, Tsai Y. Stressors of living with HIV?AIDS: patients' perspectives. *Kaohsiung Journal of Medical Science.* 2000;16(3):148-155.

57. Issiaka S, Cartoux M, Ky-Zerbo O, et al. Living with HIV: women's experience in Burkina Faso, West Africa. *AIDS care.* Feb 2001;13(1):123-128.

58. Johnson OE. Social impact of HIV/AIDS on clients attending a teaching hospital in Southern Nigeria. *SAHARA J : journal of Social Aspects of HIV/AIDS Research Alliance / SAHARA , Human Sciences Research Council.* 2012;9(2):47-53.

59. Kadowa I, Nuwaha F. Factors influencing disclosure of HIV positive status in Mityana district of Uganda. *African health sciences.* Mar 2009;9(1):26-33.

60. Kerrigan D, Bastos FI, Malta M, Carneiro-da-Cunha C, Pilotto JH, Strathdee SA. The search for social validation and the sexual behavior of people living with HIV in Rio de Janeiro, Brazil: understanding the role of treatment optimism in context. *Soc Sci Med.* May 2006;62(10):2386-2396.

61. Kilewo C, Massawe A, Lyamuya E, et al. HIV counseling and testing of pregnant women in sub-Saharan Africa: experiences from a study on prevention of mother-to-child HIV-1 transmission in Dar es Salaam, Tanzania. *Journal of acquired immune deficiency syndromes.* Dec 15 2001;28(5):458-462.

62. Klopper C, Stellenberg E, van der Merwe A. Stigma and HIV disclosure in the Cape Metropolitan area, South Africa. *African journal of AIDS research : AJAR.* 2014;13(1):37-43.

63. Kumar A, Waterman I, Kumari G, Carter AO. Prevalence and correlates of HIV serostatus disclosure: a prospective study among HIV-infected postparturient women in Barbados. *AIDS patient care and STDs.* Oct 2006;20(10):724-730.

64. Kyaddondo D, Wanyenze RK, Kinsman J, Hardon A. Disclosure of HIV status between parents and children in Uganda in the context of greater access to treatment. *SAHARA J : journal of Social Aspects of HIV/AIDS Research Alliance / SAHARA , Human Sciences Research Council.* Jul 2013;10 Suppl 1:S37-45.

65. Laryea M, Gien L. The impact of HIV-positive diagnosis on the individual, Part 1: Stigma, rejection, and loneliness. *Clinical nursing research.* Aug 1993;2(3):245-263, discussion 263-246.

66. Lee SJ, Li L, Iamsirithaworn S, Khumtong S. Disclosure challenges among people living with HIV in Thailand. *International journal of nursing practice.* Aug 2013;19(4):374-380.

67. Liamputtong P, Haritavorn N. To tell or not to tell: disclosure to children and family amongst Thai women living with HIV/AIDS. *Health promotion international.* Mar 2016;31(1):23-32.

68. Lugalla J, Yoder S, Sigalla H, Madihi C. Social context of disclosing HIV test results in Tanzania. *Culture, health & sexuality.* 2012;14 Suppl 1:S53-66.

69. Madiba S, Canti-Sigaqa V. Barriers to participate in support groups for people living with HIV: a qualitative study with men receiving antiretroviral treatment in a HIV clinic in Mthatha, South Africa. *Global journal of health science.* Nov 2012;4(6):119-128.

70. Madiba S, Letsoalo R. HIV disclosure to partners and family among women enrolled in prevention of mother to child transmission of HIV program: implications for infant feeding in poor resourced communities in South Africa. *Global journal of health science.* Jul 2013;5(4):1-13.

71. Maiorana A, Koester KA, Myers JJ, et al. Helping patients talk about HIV: inclusion of messages on disclosure in prevention with positives interventions in clinical settings. *AIDS education and prevention : official publication of the International Society for AIDS Education.* Apr 2012;24(2):179-192.

72. Maliska IC, Padilha MI, Vieira M, Bastiani J. [Perceptions and significance of being diagnosed and living with HIV/AIDS]. *Revista gaucha de enfermagem / EENFUFRGS.* Mar 2009;30(1):85-91.

73. Maman S, Mbwambo J, Hogan NM, Kilonzo GP, Sweat M. Women's barriers to HIV-1 testing and disclosure: challenges for HIV-1 voluntary counselling and testing. *AIDS care.* Oct 2001;13(5):595-603.

74. Maman S, Mbwambo JK, Hogan NM, Weiss E, Kilonzo GP, Sweat MD. High rates and positive outcomes of HIV-serostatus disclosure to sexual partners: reasons for cautious optimism from a voluntary counseling and testing clinic in Dar es Salaam, Tanzania. *AIDS and behavior.* Dec 2003;7(4):373-382.

75. Manopaiboon C, Shaffer N, Clark L, et al. Impact of HIV on families of HIV-infected women who have recently given birth, Bangkok, Thailand. *Journal of acquired immune deficiency syndromes and human retrovirology : official publication of the International Retrovirology Association.* May 1 1998;18(1):54-63.

76. Mayanja BN, Ekoru K, Namugenyi H, Lubega R, Mugisha JO. Patients' worries before starting antiretroviral therapy and their association with treatment adherence and outcomes: a prospective study in rural Uganda, 2004 - 2009. *BMC research notes.* 2013;6:187.

77. Mburu G, Hodgson I, Kalibala S, et al. Adolescent HIV disclosure in Zambia: barriers, facilitators and outcomes. *Journal of the International AIDS Society.* 2014;17:18866.

78. Musumari PM, Feldman MD, Techasrivichien T, Wouters E, Ono-Kihara M, Kihara M. "If I have nothing to eat, I get angry and push the pills bottle away from me": A qualitative study of patient determinants of adherence to antiretroviral therapy in the Democratic Republic of Congo. *AIDS care.* 2013;25(10):1271-1277.

79. Nakigozi G, Atuyambe L, Kamya MR, et al. A qualitative study of barriers to enrollment into free HIV care: Perspectives of never-in-care HIV-positive patients and providers in Rakai, Uganda. 2013:470245.

80. Nam SL, Fielding K, Avalos A, Gaolathe T, Dickinson D, Geissler PW. Discussing matters of sexual health with children: what issues relating to disclosure of parental HIV status reveal. *AIDS care.* Mar 2009;21(3):389-395.

81. Newman CE, Bonar M, Greville HS, Thompson SC, Bessarab D, Kippax SC. Barriers and incentives to HIV treatment uptake among Aboriginal people in Western Australia. *Aids.* Jan 2007;21 Suppl 1:S13-17.

82. Okoronkwo I, Okeke U, Chinweuba A, Iheanacho P. Nonadherence Factors and Sociodemographic Characteristics of HIV-Infected Adults Receiving Antiretroviral Therapy in Nnamdi Azikiwe University Teaching Hospital, Nnewi, Nigeria. *Isrn Aids.* 2013;2013:843794.

83. Olagbuji BN, Ezeanochie MC, Agholor KN, Olagbuji YW, Ande AB, Okonofua FE. Spousal disclosure of HIV serostatus among women attending antenatal care in urban Nigeria. *Journal of obstetrics and gynaecology : the journal of the Institute of Obstetrics and Gynaecology.* Aug 2011;31(6):486-488.

84. Osinde MO, Kakaire O, Kaye DK. Factors associated with disclosure of HIV serostatus to sexual partners of patients receiving HIV care in Kabale, Uganda. *International journal of gynaecology and obstetrics: the official organ of the International Federation of Gynaecology and Obstetrics.* Jul 2012;118(1):61-64.

85. Paiva V, Ayres JR, Segurado AC, et al. [The sexuality of HIV-positive adolescents: rights and challenges for healthcare]. *Ciencia & saude coletiva.* Oct 2011;16(10):4199-4210.

86. Patel SV, Patel SN, Baxi RK, et al. HIV serostatus disclosure: Experiences and perceptions of people living with HIV/AIDS and their service providers in Gujarat, India. *Industrial psychiatry journal.* Jul 2012;21(2):130-136.

87. Petrak JA, Doyle AM, Smith A, Skinner C, Hedge B. Factors associated with self-disclosure of HIV serostatus to significant others. *British journal of health psychology.* Feb 2001;6(Pt 1):69-79.

88. Phaladze NA, Human S, Dlamini SB, et al. Quality of life and the concept of "living well" with HIV/AIDS in sub-Saharan Africa. *Journal of nursing scholarship : an official publication of Sigma Theta Tau International Honor Society of Nursing / Sigma Theta Tau.* 2005;37(2):120-126.

89. Pugatch D, Bennett L, Patterson D. HIV medication adherence in adolscents. *Journal of HIV/AIDS Prevention & Education for Adolescents and Children.* 2002;5(1-2):9-29.

90. Rosen S, Ketlhapile M. Cost of using a patient tracer to reduce loss to follow-up and ascertain patient status in a large antiretroviral therapy program in Johannesburg, South Africa. *Tropical medicine & international health : TM & IH.* Jun 2010;15 Suppl 1:98-104.

91. Rothberg A, Van Huyssteen K. Employees' perceptions of the Aid-for-AIDS disease-management programme, South Africa. *African journal of AIDS research : AJAR.* Nov 2008;7(3):335-339.

92. Rujumba J, Neema S, Byamugisha R, Tylleskar T, Tumwine JK, Heggenhougen HK. "Telling my husband I have HIV is too heavy to come out of my mouth": pregnant women's disclosure experiences and support needs following antenatal HIV testing in eastern Uganda. *Journal of the International AIDS Society.* 2012;15(2):17429.

93. Schlebusch L, Vawda N. HIV-infection as a self-reported risk factor for attempted suicide in South Africa. *African journal of psychiatry.* Sep 2010;13(4):280-283.

94. Seid M, Wasie B, Admassu M. Disclosure of HIV positive result to a sexual partner among adult clinical service users in Kemissie district, northeast Ethiopia. *African journal of reproductive health.* Mar 2012;16(1):97-104.

95. Sethosa E, Peltzer K. Evaluation of HIV counselling and testing, self-disclosure, social support and sexual behaviour change among a rural sample of HIV reactive patients in South Africa. *Curationis.* Feb 2005;28(1):29-41.

96. Siegel K, Lekas HM, Schrimshaw EW. Serostatus disclosure to sexual partners by HIV-infected women before and after the advent of HAART. *Women & health.* 2005;41(4):63-85.

97. Siu GE, Bakeera-Kitaka S, Kennedy CE, Dhabangi A, Kambugu A. HIV serostatus disclosure and lived experiences of adolescents at the Transition Clinic of the Infectious Diseases Clinic in Kampala, Uganda: a qualitative study. *AIDS care.* 2012;24(5):606-611.

98. Smith MY, Rapkin BD. Social support and barriers to family involvement in caregiving for persons with AIDS: implications for patient education. *Patient education and counseling.* Jan 1996;27(1):85-94.

99. Ssali SN, Atuyambe L, Tumwine C, et al. Reasons for disclosure of HIV status by people living with HIV/AIDS and in HIV care in Uganda: an exploratory study. *AIDS patient care and STDs.* Oct 2010;24(10):675-681.

100. Stinson K, Myer L. Barriers to initiating antiretroviral therapy during pregnancy: a qualitative study of women attending services in Cape Town, South Africa. *African journal of AIDS research : AJAR.* Mar 2012;11(1):65-73.

101. Stutterheim SE, Shiripinda I, Bos AE, et al. HIV status disclosure among HIV-positive African and Afro-Caribbean people in the Netherlands. *AIDS care.* Feb 2011;23(2):195-205.

102. Syed IA, Syed Sulaiman SA, Hassali MA, Thiruchelvum K, Lee CK. A qualitative insight of HIV/AIDS patients' perspective on disease and disclosure. *Health expectations : an international journal of public participation in health care and health policy.* Dec 2015;18(6):2841-2852.

103. Terry SD, Jones JE, Brown RH. Dental-care experiences of people living with HIV/AIDS in Aotearoa New Zealand. *The New Zealand dental journal.* Jun 1994;90(400):49-55.

104. Teti M, Bowleg L, Cole R, et al. A mixed methods evaluation of the effect of the protect and respect intervention on the condom use and disclosure practices of women living with HIV/AIDS. *AIDS and behavior.* Jun 2010;14(3):567-579.

105. Thanh DC, Moland KM, Fylkesnes K. The context of HIV risk behaviours among HIV-positive injection drug users in Viet Nam: moving toward effective harm reduction. *BMC public health.* 2009;9:98.

106. Thomas B, Nyamathi A, Swaminathan S. Impact of HIV/AIDS on mothers in southern India: a qualitative study. *AIDS and behavior.* Oct 2009;13(5):989-996.

107. Titilope AA, Adediran A, Umeh C, Akinbami A, Unigwe O, Akanmu AS. Psychosocial Impact of disclosure of HIV Serostatus in heterosexual relationship at the Lagos University teaching Hospital, Nigeria. *Nigerian medical journal : journal of the Nigeria Medical Association.* Jan 2011;52(1):55-59.

108. Tzemis D, Forrest JI, Puskas CM, et al. Identifying self-perceived HIV-related stigma in a population accessing antiretroviral therapy. *AIDS care.* 2013;25(1):95-102.

109. Unge C, Sodergard B, Thorson A, et al. HIV treatment in times of civil strife: serious threats to antiretroviral drug access in the Kibera slum following the Kenyan elections. *Aids.* Aug 20 2008;22(13):1693-1694.

110. Vallerand AH, Hough E, Pittiglio L, Marvicsin D. The process of disclosing HIV serostatus between HIV-positive mothers and their HIV-negative children. *AIDS patient care and STDs.* Feb 2005;19(2):100-109.

111. Van Devanter N, Duncan A, Birnbaum J, Burrell-Piggott T, Siegel K. Gender Power Inequality and Continued Sexual Risk Behavior among Racial/Ethnic Minority Adolescent and Young Adult Women Living with HIV. *Journal of AIDS & clinical research.* Nov 25 2011(S1).

112. Van Nuil JI, Mutwa P, Asiimwe-Kateera B, et al. "Let's talk about sex": a qualitative study of Rwandan adolescents' views on sex and HIV. *PloS one.* 2014;9(8):e102933.

113. Vance DE, Woodley RA. Strengths and distress in adults who are aging with HIV: a pilot study. *Psychological reports.* Apr 2005;96(2):383-386.

114. Varni SE, Miller CT, McCuin T, Solomon SE. Disengagement and Engagement Coping with HIV/AIDS Stigma and Psychological Well-Being of People with HIV/AIDS. *Journal of social and clinical psychology.* Feb 1 2012;31(2):123-150.

115. Visser MJ, Neufeld S, de Villiers A, Makin JD, Forsyth BW. To tell or not to tell: South African women's disclosure of HIV status during pregnancy. *AIDS care.* Oct 2008;20(9):1138-1145.

116. Walker S. *Vertically transmitted HIV+/AIDS: The impact on maternal attachment.* Dissertation Abstracts International: Section B: The Sciences and Engineering, Vol 57(3-B). pp.2169. 2012.

117. Ware NC, Wyatt MA, Tugenberg T. Social relationships, stigma and adherence to antiretroviral therapy for HIV/AIDS. *AIDS care.* Nov 2006;18(8):904-910.

118. Webel AR. Testing a peer-based symptom management intervention for women living with HIV/AIDS. *Aids Care-Psychological and Socio-Medical Aspects of Aids/Hiv.* 2010;22(9):1029-1040.

119. Wolf HT, Halpern-Felsher BL, Bukusi EA, Agot KE, Cohen CR, Auerswald CL. "It is all about the fear of being discriminated [against]...the person suffering from HIV will not be accepted": a qualitative study exploring the reasons for loss to follow-up among HIV-positive youth in Kisumu, Kenya. *BMC public health.* 2014;14:1154.

120. Zhou Y, Zhang L, Li X, Kaljee L. Do Chinese parents with HIV tell their children the truth? A qualitative preliminary study of parental HIV disclosure in China. *Child: care, health and development.* Nov 2013;39(6):816-824.

121. Zukoski AP, Thorburn S, Stroud J. Seeking information about HIV/AIDS: a qualitative study of health literacy among people living with HIV/AIDS in a low prevalence context. *AIDS care.* Nov 2011;23(11):1505-1508.
